# Stereology as the 3D tool to quantitate lung architecture

**DOI:** 10.1007/s00418-020-01927-0

**Published:** 2020-10-13

**Authors:** Lars Knudsen, Christina Brandenberger, Matthias Ochs

**Affiliations:** 1 Institute of Functional and Applied Anatomy, Hannover Medical School, Hannover, Germany; 2 Biomedical Research in Endstage and Obstructive Lung Disease Hannover (BREATH), German Center for Lung Research (DZL), Hannover, Germany; 3 REBIRTH Cluster of Excellence, Hannover, Germany; 4 Institute of Functional Anatomy, Charité-Universitätsmedizin Berlin, Philippstr. 11, 10115 Berlin, Germany; 5 German Center for Lung Research (DZL), Berlin, Germany

**Keywords:** Lung, Stereology, Volume electron microscopy, Laser scanning microscopy, Micro-computed tomography

## Abstract

Stereology is the method of choice for the quantitative assessment of biological objects in microscopy. It takes into account the fact that, in traditional microscopy such as conventional light and transmission electron microscopy, although one has to rely on measurements on nearly two-dimensional sections from fixed and embedded tissue samples, the quantitative data obtained by these measurements should characterize the real three-dimensional properties of the biological objects and not just their “flatland” appearance on the sections. Thus, three-dimensionality is a built-in property of stereological sampling and measurement tools. Stereology is, therefore, perfectly suited to be combined with 3D imaging techniques which cover a wide range of complementary sample sizes and resolutions, e.g. micro-computed tomography, confocal microscopy and volume electron microscopy. Here, we review those stereological principles that are of particular relevance for 3D imaging and provide an overview of applications of 3D imaging-based stereology to the lung in health and disease. The symbiosis of stereology and 3D imaging thus provides the unique opportunity for unbiased and comprehensive quantitative characterization of the three-dimensional architecture of the lung from macro to nano scale.

## Stereology of the lung and classical microscopy techniques

### The “dimension trap” in microscopy and its solution by stereology

Many optical illusions are based on our misinterpretation of two-dimensional figures as representations of three-dimensional objects. And although we are aware of this problem, they still work. In the context of microscopy, there is a related problem regarding the misinterpretation of what we see. This pertinacious problem may be termed the “dimension trap”. To get insight into the fine structure of three-dimensional biological objects, the classical approach is to use microscopes to investigate nearly two-dimensional sections produced by microtomes from small samples taken from these objects. Conventional light and transmission electron microscopy (TEM) rely on this principle. However, as a result of this sectioning process, we lose one dimension, and therefore we lose important qualitative and quantitative information. And although we are (or at least should) be aware of this loss of information, it is still commonly overlooked, as indicated by many published studies containing incorrect data because this “dimension trap” is ignored (for a survey in lung research, see Mühlfeld et al. 2015).

There is, however, a solution to this problem: stereology. Based on mathematical principles coming from the field of stochastic geometry, stereology was developed as a set of methods to obtain quantitative three-dimensional information based on measurements on properly sampled nearly two-dimensional microscopic sections. As such, three-dimensionality is a built-in property of stereology, as opposed to “planimetric” image analysis of pixels which ignores the “dimension trap”. Thus, stereology is not just one but actually THE 3D technique for quantitative microscopy (for introduction to the foundations and practical applications, see Baddeley and Vedel Jensen 2005 and Howard and Reed 2005).

A pioneer in the development of stereology was Hans Elias (1907–1985). Elias, a German of Jewish descent who later became a US citizen, was a man with broad interests both in science and art. He studied fine arts as well as mathematics, biology and physics and worked as a painter and sculptor, but also as teacher, a cinematographer, and finally as an anatomist (for details, see Hildebrandt 2012). Particular hallmarks of Elias´ scientific oeuvre were the brilliant stereograms drawn by him which illustrated the three-dimensional architecture of histologic structures (see e.g. Elias 1972; Elias et al. 1978). In his publications, Elias emphasized the pitfalls of the three-dimensional interpretation of microscopic sections (e.g. Elias 1949, 1971). His motivation was to overcome these pitfalls by combining common sense and a “feel” for three-dimensional space with rigorous methods for quantification. He was the driving force for the foundation of the International Society for Stereology at a small meeting organized by him on the Feldberg mountain in the Black Forrest on May 11th and 12th, 1961. At this meeting, Elias also coined the term stereology (Greek stereos = solid, spatial). Elias defined stereology as “extrapolation from two- to three-dimensional space, or three-dimensional interpretation of two-dimensional images, by methods of geometric probability” (Elias 1971). He also pointed out that “the term stereology was coined to describe simply our interest in three-dimensional space” (Elias et al. 1971). Thus, from its very beginning stereology was conceived as a science to study the three-dimensional properties of objects.

**Fig. 1:**
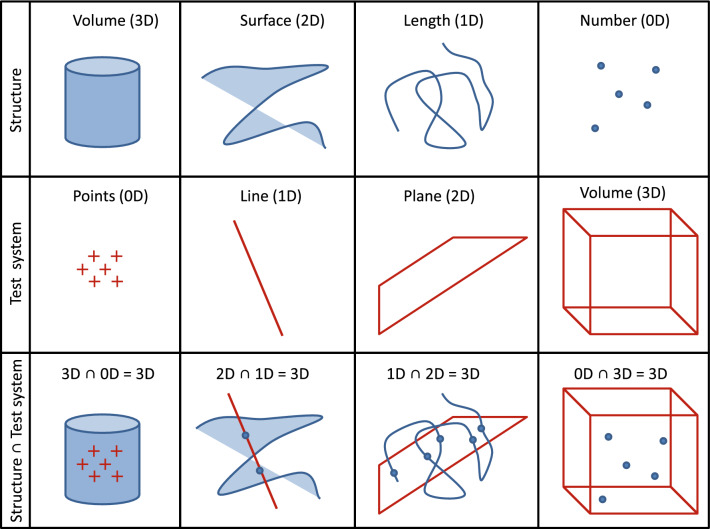
Three-dimensionality in stereology. Illustration of the relationships between structure parameters, suitable test systems and their interactions (which create counting events, the basic units of measurement in stereology). Note that, for each parameter, the sum of the dimension of the structure parameter and the dimension of the test system always equals three. Therefore, test points “feel” volume, test lines “feel” surface area, and test planes “feel” length. Only test volumes (i.e. disectors) can “feel” particle number. Figure modified from Brandenberger et al. 2015

How does stereology take three-dimensionality in “flatland” microscopy into account? Using appropriate and very simple test systems for measurement (see Fig. 1). These test systems (e.g. sets of points or lines or planes) are superimposed over microscopic images and interact with structures contained in these sectional images, thus creating events that can be counted (e.g. test points “hit” structure profile area or test lines “hit” structure boundary or test planes “hit” structure transsect). The important mathematical principle is that the dimension of the test system is adjusted to the dimension of the parameter of interest (e.g. volume or surface area or length) to sum up to three. Thus, test points (zero-dimensional) are used for volume estimation (three-dimensional), test lines (one-dimensional) are used for surface area estimation (two-dimensional) and test planes (two-dimensional) are used for length estimation (one-dimensional). A special case is number estimation (zero-dimensional), because it requires a three-dimensional test system. As the quantitative information about particle number in a three-dimensional reference volume is not contained in single thin (nearly two-dimensional) microscopic sections, it is not possible to estimate number when only those sections are available. In stereology, this problem was solved with the disector (Sterio 1984). A disector can be defined as a three-dimensional test system (i.e. a test volume), the only test system that contains the “tops” of particles which can be used to count them independent of their size or their spatial orientation to the section plane. In conventional light microscopy and TEM, disectors can be created by producing two physical sections from the same tissue block with a known distance (hence the name: two sections = di-sector). An overview of stereological terms used in this review is provided in Table 1.

**Table 1:** Stereological terms and their definition

Stereological term	Definition
Cavalieri method	An unbiased approach for estimating the volume of an object by thoroughly slicing it in parallel sections of a known thickness and estimating the area of the cut surfaces with a point grid. The volume is calculated by multiplying the total surface area with the section thickness
Connectivity	Describes the number of units in a network and can be estimated based on the topological Euler-Poincaré characteristics (Euler number *χ*) in combination with the disector principle
Disector (physical or optical)	A 3D stereological test system (test volume) for sampling and counting objects. By either using two sections (physical disector) or by focussing through one thick section in z direction (optical disector), a known z distance with a defined counting area is sampled, and particle number and connectivity in 3D space can be estimated
Fractionator	An unbiased sampling method that is based on keeping track of sampling fractions at each subsampling step to obtain total values by multiplying the counted objects at the final sampling step with the inverse of the sampling fraction. The method can optimally be combined with the optical disector (optical fractionator)
Isector	A method for generating isotropic uniform random (IUR) sections by embedding the tissue in spherical molds to randomize orientation in further tissue processing
Nucleator	A disector-based number-weighted local particle size estimator (compare point-sampled intercepts). Particles are first sampled with the disector (i.e. in proportion to their number). Then, their volume is estimated by measurements towards the particle boundaries. Alternative to rotator
Orientator	A method for generating isotropic uniform random (IUR) sections via a two-step process, including randomized dis-orientation along two different axes
Point-sampled intercepts	A single section-based volume-weighted local particle size estimator (compare nucleator and rotator). Particles are first sampled with test points (i.e. in proportion to their volume). Then, their volume is estimated by measurements towards the particle boundaries. Used to estimate star volume
Reference volume	The space from which samples are taken and in which particular stereological measurements are performed, e.g. total lung volume. Knowledge of the reference volume is crucial to convert densities estimated with stereological test systems to total quantities
Rotator	A disector-based number-weighted local particle size estimator (compare point-sampled intercepts). Particles are first sampled with the disector (i.e. in proportion to their number). Then, their volume is estimated by measurements towards the particle boundaries. Alternative to nucleator
Star volume	An estimator for calculating the volume-weighted mean volume of an object using point-sampled intercepts
Systematic uniform random sampling (SURS)	An unbiased sampling method that includes a systematic and a random component, by sampling with a constant interval and beginning with a random starting point
Unbiased counting frame	A stereological test plane to count the profiles or transsects of objects within a defined area. It consists of a frame with two inclusion and two exclusion ("forbidden") lines and their extensions to avoid over- or underestimation of the counts

The introduction of the disector principle in 1984 (Sterio 1984) marked a watershed in the history of stereology. On the one hand, it completed the set of test systems for the estimation of first order stereological parameters (volume, surface area, length and, finally, number). On the other hand, it extended stereology from the analysis of single, nearly two-dimensional microscopic sections into analysis of three-dimensional microscopic datasets. The disector is therefore of particular interest in the context of 3D imaging techniques (see below).

The development and application of stereology to the lung was pioneered by Ewald Weibel (1929–2019) (Weibel 1963, see also Ochs 2020). Stereology also became the basis for an official research policy statement on quantitative assessment of lung structure, published on behalf of the American Thoracic Society and the European Respiratory Society (Hsia et al. 2010). While this document described the state of the art in the application of stereological methods to study the lung based on classical light microscopy and TEM, it also pointed out the great potential of the combination of stereology with in vivo lung imaging techniques where stereology can directly be applied to three-dimensional imaging datasets. But vice versa, the rapidly emerging field of 3D lung imaging should also be aware of the concepts and tools that stereology has to offer.

## Stereology of the lung and 3D imaging techniques

### The challenges and limits of two-dimensional techniques

In the last decades design-based stereology in the lung has predominantly been performed using physical, two-dimensional sections for classical light microscopy or TEM. Generating accurate and precise morphometrical data such as surface areas available for gas exchange, thickness of the blood-gas barrier or the number of alveoli per lung, the foundation for the establishment of pulmonary structure–function relationships was thereby provided both for healthy and diseased lungs (Weibel et al. 1992, 1993, 2007; Weibel 2017; Hsia et al. 2010). The determination of numbers of defined anatomical structures within the lung, such as alveolar epithelial type II cells or on larger scale alveoli and complete acini, became feasible in an unbiased manner with the development of the physical disector method using “classical” microscopic (i.e. basically two-dimensional) sections. The physical disector method is based on generating a three-dimensional test volume for counting of profiles of anatomical structures by two (or more), often consecutive, parallel two-dimensional sections characterized by a known surface area of the counting frame which is projected on the sections as well as a known distance between these sections (Sterio 1984; Jung et al. 2005; Ochs et al. 2004a; Wulfsohn et al. 2010; Hyde et al. 2004). However, counting defined anatomical structures based on the physical disector method using two-dimensional, physical pairs of sections at light or electron microscopical level is technically demanding and time consuming. As a consequence the application of these methods in biomedical research of the respiratory system has been shown to be relatively limited (Mühlfeld et al. 2015). In addition, the use of physical two-dimensional sections for design-based stereology at light or electron microscopic level to extensively characterize complex 3D structures such as the conducting airways, the acini as functional unit of the lung (Wulfsohn et al. 2010) or the capillary network within the interalveolar septa (Mühlfeld et al. 2010) is challenging and limited.

### Counting and sampling in 3D: The optical disector

Hence, the development of three-dimensional, volumetric imaging techniques generating digital stacks of nearly two-dimensional images of a known thickness (these images herein after referred to as slices) in combination with design-based stereological methods provided the basis to determine unbiased data of certain structural features in the lung in a more comprehensive way than the classical physical disector approach. In this regard, three-dimensional datasets in which the investigators can scroll through the stack of two-dimensional images turned out to be a powerful approach in design-based stereology to investigate important features of the acini, e.g. mean acinar size or total number of acini per lung (Barré et al. 2014; Haberthür et al. 2013; Barré et al. 2016; McDonough et al. 2011; Vasilescu et al. 2020; Verleden et al. 2020). The structure and function of the alveolar capillary network follow the sheet-flow concept so that it consists of a network of capillary segments whose diameters are larger than the lengths (Fung and Sobin 1969; Mühlfeld et al. 2010). Using classical two-dimensional imaging methods it is possible to determine the surface area of the capillary network or its volume. The use of three-dimensional datasets moreover enabled stereologists to determine also the connectivity of the alveolar capillary network (Grothausmann et al. 2017; Mühlfeld et al. 2018; Buchacker et al. 2019; Willführ et al. 2015). The connectivity describes the topological complexity of a structure in two or three dimensions, based on the so-called Euler-Poincaré characteristics (Kroustrup and Gundersen 2001; Boyce et al. 1995; Gundersen et al. 1993). In the context of the alveolar capillary network the Euler-Poincaré characteristic provides the number of capillary segments / loops (Willführ et al. 2015), a parameter which has been suggested to be of interest in lung development and developmental disorders such as broncho-pulmonary dysplasia (Mühlfeld et al. 2018).

Taken together, the huge advantage of three-dimensional datasets is the fact that they can be combined with the “optical” disector principle, a stereological tool to determine numbers or the Euler-Poincaré characteristic of structures of interest within unbiased and randomized test volumes (Sterio 1984; Bjugn and Gundersen 1993; Vanhecke et al. 2007). The basic concept behind the “optical” disector is to scroll the focus plane combined with an unbiased counting frame of a known area along the third dimension (e.g. the *z*-axis) through the sampled tissue. Each time a structure of interest (e.g. a cell) comes into focus for the first time and this profile is located within the unbiased counting frame (not touching the "forbidden" line, see Gundersen 1977) it is counted (= counting event). When the depth in the third dimension along which the area of the counting frame has been moved is known, the volume in which the counting events of the structure of interest were recorded can be used for calculation as densities, e.g. number of cells per volume of lung tissue. This numerical density can be converted into a total number per organ. This last step, however, requires the determination of the size of the reference space, e.g. the volume of the lung. In the past this method has mainly been used for confocal microscopy where the optical focus plane with a thickness of a few hundred nanometers is moved through the tissue so that it has accordingly been termed “optical disector” (West et al. 1991; Gundersen et al. 1988). Since cells might be very complex structures in 3D (like the alveolar epithelial type I cell in the lung (Schneider et al. 2019)) it has turned out to be beneficial to count the nucleus (or nucleolus) within the cell, in particular if confocal microscopy is used for scrolling the optical focal plane through a thick tissue sample (Schipke et al. 2014; Kubínová and Janáček 2015; West et al. 1991). An alternative approach of counting particles in an organ is the so-called fractionator sampling in combination with the disector. The basic concept behind this approach is that the number of the structure of interest is counted within a well-known sampling fraction of the complete organ (e.g. 1/1,000,000) so that the number of the structure in the complete organ can be calculated by multiplication of the number counted with the inverse of that sampling fraction (Gundersen 2002, 1986; Knust et al. 2009; Jansing et al. 2018). One major advantage of the fractionator is due to the fact that number estimation based on this sampling principle is independent of tissue deformation. The fractionator can be optimally combined with the optical disecor by considering the sampling fraction in all three dimensions. If the counting is performed by scrolling the sectional plane (e.g. optical focus plane in confocal microscopy) through the three-dimensional dataset and the relative sampling fraction of the tissue depth is known and used to calculate the total number of the structure of interest, this method can be termed “optical fractionator” (Dorph-Petersen et al. 2001; Jansing et al. 2018).

The “optical” disector principle can not only be used to count structures, but also to sample structures in 3D in an unbiased way, e.g. independent of their size, shape or orientation. This is crucial to determine the so-called number-weighted mean volume of the structures of interest. Hence, the combination of the optical disector (used for sampling) with local stereological probes such as the nucleator or rotator can be applied to estimate the individual size of the sampled structures and therefore the number-weighted mean volume of the population of the structures of interest within the organ (Gundersen 1988; Møller et al. 1990; Tandrup et al. 1997; Rasmusson et al. 2013). Another option to determine the volume of sampled anatomical structures in three-dimensional datasets is the so-called Cavalieri principle (Gundersen and Jensen 1987). The structure of interest is virtually cut in slices of equal thickness. A two-dimensional point grid is randomly projected on the cut surface of each slice and points hitting this surface are counted. Multiplication of the area associated with each test point by the thickness of the slice results in the volume per test point so that the multiplication of the number of test points by the volume per test point results in the volume of the structure of interest. This method could be applied to the whole lung using computed tomography but also to individual acini which were sampled in micro-computed tomography (µCT) datasets by the “optical” disector principle and segmented (Vasilescu et al. 2012a).

### 3D imaging methods in lung research

**Fig. 2:**
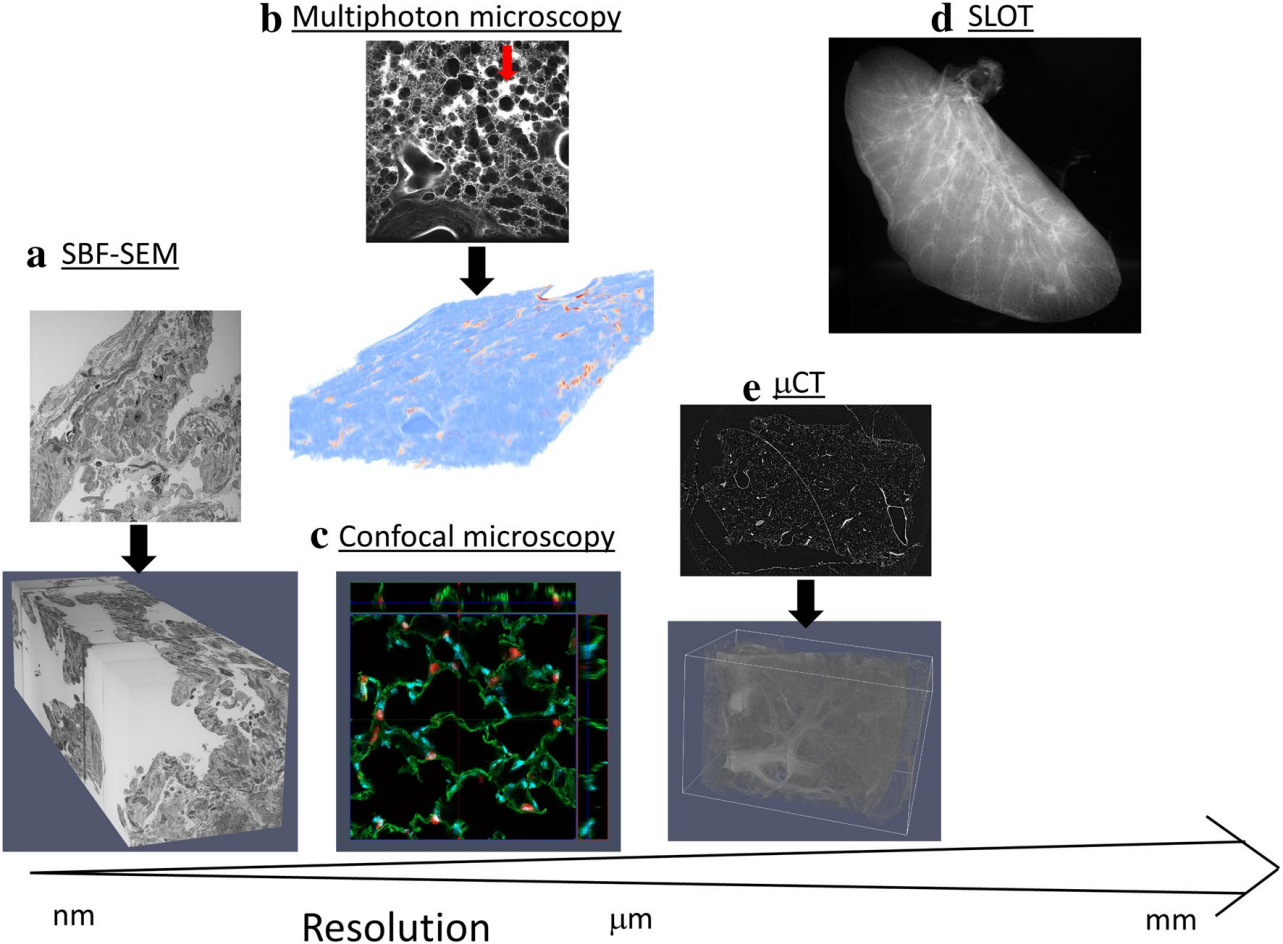
Examples of three-dimensional datasets. **a** Serial block face scanning electron microscopy (SBF-SEM) as one example for volume EM is based on a registered stack of automatically generated two-dimensional SEM images. Here, a sample of a lung explant from a patient suffering from idiopathic pulmonary fibrosis is illustrated. This sample has been taken from material processed within the frame of a previous study (Lutz et al. 2015). **b** A stack of virtual, two-dimensional images was generated using multi-photon microscopy to create a three-dimensional dataset. This lung was part of a study of ventilation-induced lung injury (Albert et al. 2020), using intravenous administration of Evans blue which binds to albumin so that those areas suffering from vascular leakage could be identified (arrow). **c** A confocal microscopic image shows a 3D view of the lung parenchyma with alveolar epithelial type II cells labeled in red, cell nuclei in blue and septa in green. In this specimen, tamoxifen treatment of Sftpc-Cre(ERT2)/ROSAtdTomato mice resulted in red fluorescent tdTomato positive alveolar epithelial type II cells. Pulmonary tissue sections were further stained with WGA-FITC (green) and DAPI (blue) and imaged as a 3D stack with confocal microscopy. **d** Scanning laser optical tomography (SLOT) of a left mouse lung allowing three-dimensional investigation of internal structures, such as the conducting airways down to the terminal bronchioles. This lung was part of a previous study using SLOT to study the branching pattern of the conducting airways (Funke et al. 2016). **e** Micro computed tomography (µCT) of a mouse lung. This dataset has been used in a previous publication to investigate the pulmonary vasculature in detail (Mühlfeld et al. 2018)

Figure 2 illustrates examples of three-dimensional datasets whose resolution ranges from the nanometer to millimeter scale which have been applied to the lung and can be combined with design-based stereology. These three-dimensional imaging techniques offer a spectrum of complementary sample sizes and resolutions. Array tomography, serial block face scanning EM (SBF-SEM), focused ion beam scanning EM and electron tomography have significantly advanced the field by generating three-dimensional datasets at a scale of micrometers to nanometers (Ochs et al. 2016; Schneider et al. 2020) while confocal and multi-photon microscopy are very useful to obtain such three-dimensional datasets with a resolution in the micrometer range (Kubínová and Janáček 2015). Based on the intrinsic auto-fluorescence of the lung and the transmission properties for the laser beam, scanning laser optical tomography (SLOT) has been developed and applied to investigate the internal structures of the lung in three dimensions in a non-destructive manner with a resolution sufficient to resolve alveoli but also to identify the terminal bronchioles and thus the entrance into the acinus (Kellner et al. 2012, 2015; Funke et al. 2016). With an even higher resolution of a few micrometers, µCT as well as synchrotron radiation-based X-ray tomographic microscopy represent established and powerful imaging techniques for quantitative morphometry when combined with design-based stereology (Vasilescu et al. 2013, 2020; Barré et al. 2014, 2016; Haberthür et al. 2013).

### Prerequisites: sampling, tissue deformation, spatial orientation

However, it needs to be pointed out that pitfalls and sources of bias must be avoided for all imaging techniques alike and independent of whether or not the datasets are three- or two-dimensional (Tschanz et al. 2014a). This means that tissue sampling must be representative for the whole lung to obtain representative data. In other words every part of the lung must have the same chance of being included in the stereological analyses. Methods to obtain representative sets of lung tissue are, among others, the systematic uniform random sampling principle as well as the so-called smooth fractionator sampling principle (Tschanz et al. 2014a; Gundersen 2002; Cruz-Orive and Weibel 1981). Both of these sampling procedures can be performed by slicing the lung physically (Tschanz et al. 2014a) or virtually (Vasilescu et al. 2013). The relevance of appropriate and unbiased sampling in lung diseases has been shown recently. Verleden et al. applied a multiresolution imaging approach consisting of a multidetector computed tomography (MDCT) imaging of whole lung explants in an initial step followed by µCT imaging of sampled, smaller parts of the lung (Verleden et al. 2020). Regarding the sampling procedure the investigators followed two different protocols: a random sampling where every part had the same chance of being investigated and a targeted sampling, differentiating lung regions according to the severity of disease manifestation based on the appearance in MDCT. Those parameters which were calculated up to the organ scale such as the number of terminal bronchioles diverged considerably between random sampling and targeted sampling.

As for “classical” histological tissue processing, tissue deformation must not be neglected if three-dimensional datasets are used for morphometry based on design-based stereology (Dorph-Petersen et al. 2001; Schneider and Ochs 2014). Hence, investigators using µCT datasets for design-based stereology repetitively measured the lung volume during tissue processing, including critical point drying to keep track of shrinkage which was, however, not observed during the drying process (Barré et al. 2016; Vasilescu et al. 2012b).

As pointed out above, the application of three-dimensional datasets in the context of design-based stereology is particularly beneficial for the determination of numbers or connectivity of complex structures by means of the “optical” disector principle. Moreover, three-dimensional datasets can be used to determine surface areas of interfaces (e.g. the surface area of the alveoli) by counting intersections of a (linear) test line with the surface or the length of structures (e.g. vessels) by counting profiles within a counting frame of a known area. In this regard it is essential to guarantee that the interaction of the stereological probe (e.g. test line) and the anatomical structure (e.g. surface area of alveoli) is random and dependent on the surface area density but not on the orientation within the organ. If the structure of interest, i.e. the surface area, has a random geometrical orientation in all directions of space, the structure is isotropic so that the orientation of stereological test systems can be chosen arbitrarily. If there is a preferred spatial orientation and therefore anisotropy of the anatomical structure it is essential to randomize the sectional plane through the tissue and / or the stereological test system in all three dimensions. This general rule is valid for physical two-dimensional sections used for stereology but also for three-dimensional datasets and inevitable to avoid a bias. In this regard, the spatial orientation of the section plane can be randomized by means of the ortientator (Mattfeldt et al. 1990) or isector (Nyengaard and Gundersen 1992). Three-dimensional datasets also allow the use of spatial grids for volume, surface and length estimation. The prerequisites for an unbiased determination of these stereological parameters are similar to measurements on two-dimensional sections: the spatial grid must be isotropic random in orientation and random in its position within the three-dimensional dataset. Examples of spatial grids for determination of surface area are the Fakir method which uses linear test lines orientated in all the dimensions of space (Kubínová and Janácek 1998) and the virtual cycloid method characterized by six cycloidal arcs (= spider probe) (Gokhale et al. 2004). The advantage of these spatial test systems is that they can be applied to arbitrary orientated three-dimensional datasets. In the healthy lung the surface areas of the conducting airways or the pleura are anisotropic while the alveolar surface area can be considered to be isotropic (Hsia et al. 2010).

### Applications of 3D imaging-based stereology to the lung: volume electron microscopy

**Fig. 3:**
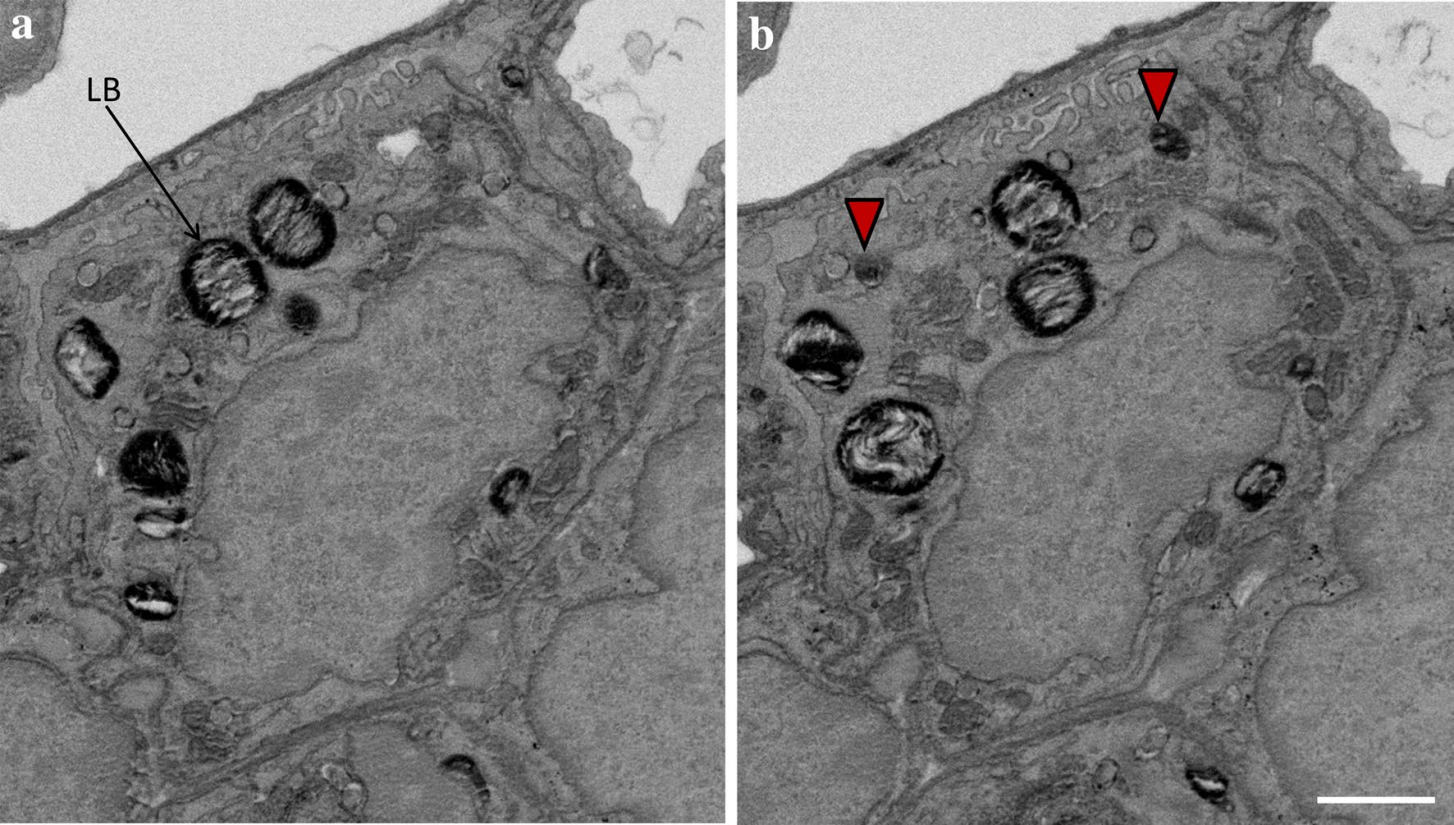
Counting of lamellar bodies (LB) using volume electron microscopy. Images were extracted from the SBF-SEM 3D data stack visualized in Fig. 2a and show an alveolar epithelial type II cell in two consecutive slices to illustrate the counting event. Based on the optical disector principle the investigator scrolls through the 3D data set and each time a LB comes into focus it is counted. In this example **a** is above from **b** so that those LBs which can be seen in **b** but not in **a** are counted (red arrowheads). The thickness of each slice is 80 nm so that the distance from one slice of the stack to the next one is 80 nm. If one imagines that the sectional plane is scrolled through the stack it becomes clear that the top of these two LBs must be located in the volume of the alveolar epithelial type II cells through which the sectional plane was swept. The volume (stereological probe) for counting of LBs (or better the top of the LB, which is independent of LB size!) is created by the height of the optical disector (here 80 nm) and the surface area of the profile of the alveolar epithelial type II cells. Since the borders of the alveolar epithelial type II cells can completely be seen it is possible to determine the cut surface area. Since two LBs come into focus within this volume, the numerical density of LB, e.g. number per volume unit of alveolar epithelial type II cells, can be determined. These densities can be transferred into total numbers per alveolar epithelial type II cell if the cell volume is known. Volumetric imaging could increase the efficiency of counting e.g. LBs or other sub-cellular structures. Scale bar = 1 µm

In the following section we describe examples of how three-dimensional datasets could be (or have already been) used in design-based stereology. As pointed out above, the strength of three-dimensional datasets is the application of the “optical” disector principle to determine numbers or connectivity based on the Euler-Poincaré characteristic of anatomical structures. At the EM level the physical disector has been used to determine the number of lamellar bodies per alveolar epithelial type II cell. Lamellar bodies are the surfactant storing organelles and the quantification of these organelles has been shown to be of relevance in mouse models with genetically modified genes of surfactant proteins or lipid transporters such as Abca3 (Ochs et al. 2004b; Jung et al. 2005; Beers et al. 2017) or lung injury (Knudsen et al. 2011; Fehrenbach et al. 1998). In these previous studies consecutive ultrathin sections of a known thickness (e.g. 90 nm) were cut and placed side by side on one grid. Images of corresponding profiles of alveolar epithelial type II cells on these pairs of ultrathin sections were taken and represented a disector pair. These disector pairs were compared with each other and profiles of lamellar bodies which were present on one but not the other section were counted. Using array tomography, SBF-SEM (Fig. 2a) or FIB-SEM stacks of two-dimensional EM images create three-dimensional datasets which all have appropriate resolution to count lamellar bodies based on the “optical” disector principle (Ochs et al. 2016; Beike et al. 2019; Buchacker et al. 2019). Figure 3 shows an extract from a three-dimensional data stack taken from the SBF-SEM dataset from Fig. 2a to illustrate the appearance of lamellar bodies as counting events. Profiles of the same alveolar epithelial type II cell are shown here and the distance from the top of the left to the top of the right section is 80 nm. To obtain unbiased data, the sampling of cells must be random. Although SBF-SEM can easily be used to generate “optical” disectors, its application in this context is limited since it is not a high throughput method, it is cost- and time-intensive and the availability is restricted. Representative sampling is critical in design-based stereology and in this regard a general rule says “do more less well” (Gundersen and Osterby 1981). This rule indicates that it is more efficient to increase the precision of the stereological data on the organ scale to include more datasets and count less per dataset instead of including only a few datasets and investigate these intensively. SBF-SEM creates high resolution datasets which in principle allow counting all lamellar bodies within one alveolar epithelial type II cell (e.g. a few large disector volumes). Based on the rule “do more less well” such an approach cannot be recommended. It is indeed preferable to investigate many smaller disector volumes (e.g. 100–200) distributed over a higher number of randomly sampled datasets. These aspects need to be taken into consideration during the planning phase of a stereological study. For this purpose it might indeed be beneficial in terms of efficiency, costs and data handling to make use of the classical, TEM-based physical disector instead of three-dimensional SFB-SEM datasets. However, as proposed by Ferguson et al. in 2017, in combination with stereological sampling principles the work-load and huge data volumes created by SBF-SEM can substantially be reduced (Ferguson et al. 2017).

### Applications of 3D imaging-based stereology to the lung: confocal microscopy

**Fig. 4:**
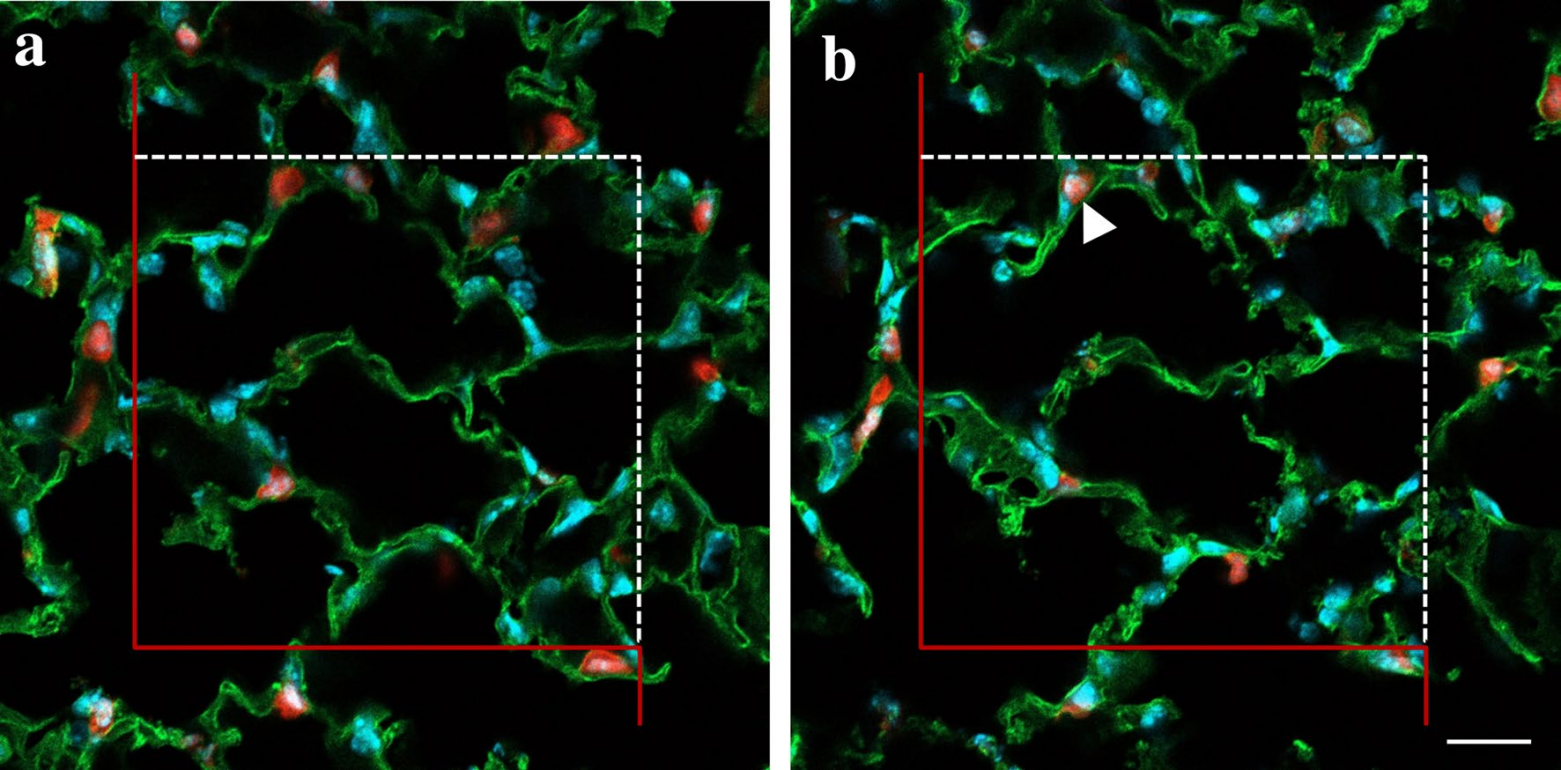
Counting red fluorescent alveolar epithelial type II cells using the optical disector on 3D confocal microscopic image stacks. The images have been extracted from the confocal microscopic image stack shown in Fig. 2c. In this stack, the image in **a** is above the image in **b**. A counting frame with an area of 120 × 120 µm^2^ is superimposed on the images and has a red forbidden line. The distance between the two virtual slices (i.e. the disector height) is 5 µm. The alveolar epithelial type II cells of tamoxifen treated Sftpc-Cre(ERT2)/ROSAtdTomato mice express the red fluorescent tdTomato protein. The nuclei are stained in blue and the nuclei of the alveolar epithelial type II cells represent the counting event. Based on the optical disector the focus plane is swept through the thick slice and each time a nucleus within an alveolar epithelial type II cell occurs within the counting frame and does not touch its forbidden line (red) it is counted. A counting event is indicated with a white arrow head. The area of the counting frame and the disector height are used to calculate the volume in which was counted. Scale bar = 20 µm

Three-dimensional imaging methods which are more prevalent in biomedical science are confocal microscopy and µCT. Confocal microscopy can be applied to specimens with a depth of several 10 µm to obtain a registered stack of serial optical slices of a thickness of approximately 200 nm or more. These image stacks can be used to quantify number or connectivity of cellular or sub-cellular structures in the lung. Figure 2c illustrates a confocal microscopic three-dimensional dataset with red fluorescent alveolar epithelial type II cells in the lung parenchyma. The number of the alveolar epithelial type II cells in the lung tissue can be estimated using the optical disector as shown in Fig. 4. Here, two slices with a height difference of 5 µm have been extracted from an optical image stack, and a counting frame with a known area was superimposed. The nuclei of the alveolar epithelial type II cells represent the counting event. A counting event occurs if a nucleus comes into focus within the counting frame (according to the unbiased counting rule, see Gundersen 1977) while scrolling through the image stack. As in every stereological approach, it is indispensable that the images/stacks were acquired randomly. To estimate the total number of cells per lung, the disector principle could be combined with the fractionator as shown by Jansing et al. (Jansing et al. 2018) or by calculating the density of cells per sampled test volume and multiplying it with the total lung volume as described for the physical disector principle (Mühlfeld and Ochs 2013; Brandenberger et al. 2015).

Confocal microscopy in combination with the optical disector has also been applied to estimate the distribution of macrophage subpopulations in the human lung (Hume et al. 2020). In their work, Hume and colleagues quantified the localization of lung macrophages in the upper right lobe of smokers and non-smokers, who presented with a clear x-ray and died from nonpulmonary causes, using design-based stereology and confocal microscopy. In the healthy human right upper lobe they estimated 2.1 billion interstitial macrophages and 78% of them were located within the interalveolar septa, while the rest could be found in the connective tissue surrounding vessels and conducting airways. Furthermore, the number and density of interstitial macrophages was 36–56% higher in smokers. The identification of preferred localization can be done using a stratified sampling approach (Nyengaard and Gundersen 2006) that includes several compartments of interest such as alveolar airspace lumen, alveolar septal tissue, conducting airways or vessels. Another advantage of confocal microscopy, besides 3D image information, is the use of immunofluorescence to identify cell types and cellular subpopulations with established markers.

**Fig. 5:**
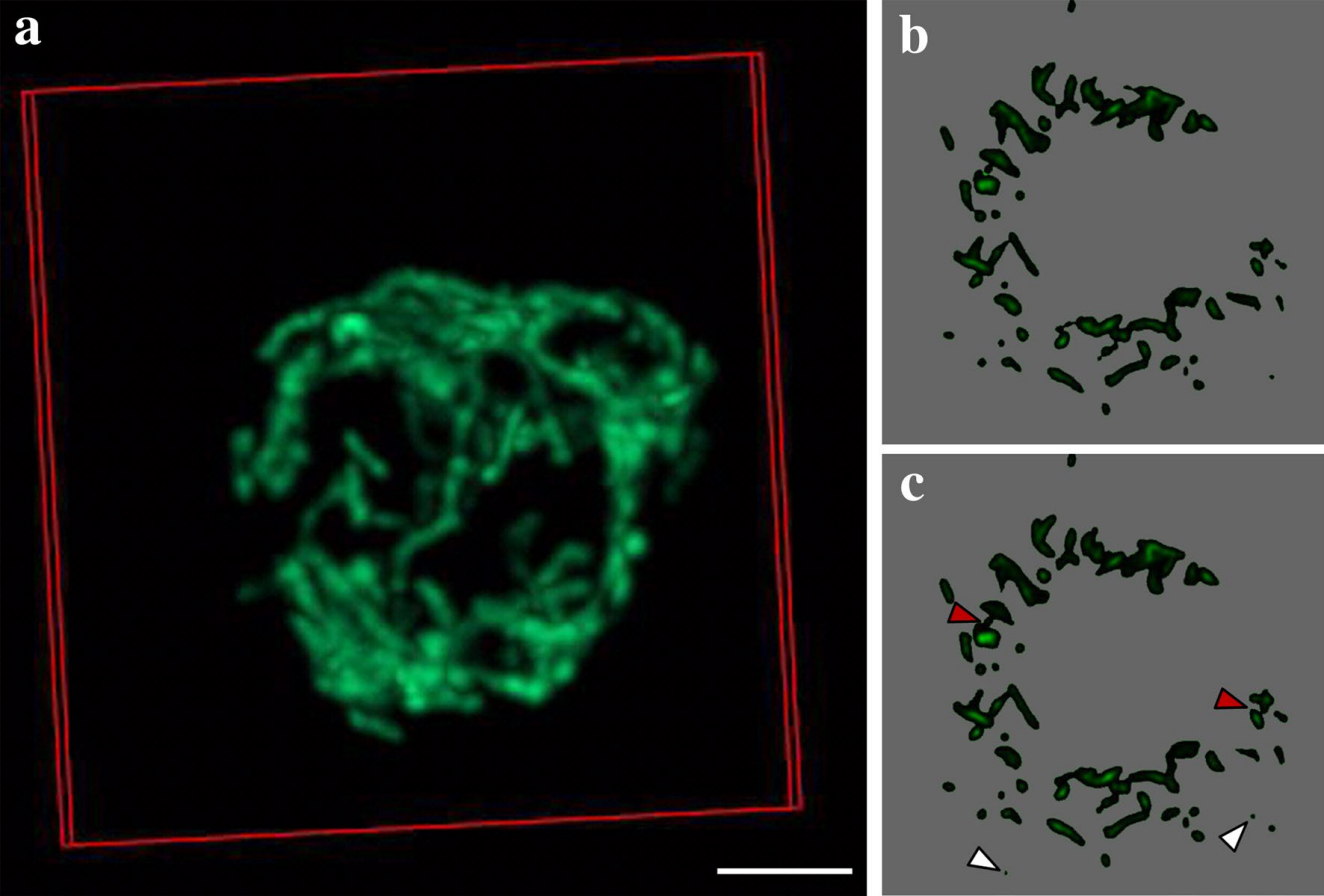
Mitochondrial connectivity estimation based on the Euler-Poincaré characteristic. The mitochondria of isolated murine macrophages were stained with JC-1 dye and imaged with a confocal LSM. Image stacks were acquired with a 60 × objective and the entire cell was imaged with a z-stack interval of 150 nm. The acquired image stack was further processed with image deconvolution (Huygens SVI) and is shown in 3D in the overview image (**a**). For the estimation of total connectivity, consecutive virtual slices were compared and analyzed for holes (H), bridges (B) and islands (I) to calculate the Euler number *χ*_3_ = I – B + H. The images in **b** and **c** show an exemplary pair of consecutive virtual slices of this image stack, where islands are marked with white arrowheads and bridges with red arrowheads. Holes are relatively rare and not seen here. Scale bar = 3 µm

Confocal microscopy, however, can also be used to quantify sub-cellular structures, if images were acquired with sufficient resolution. An example is the connectivity of mitochondria in a cell as shown for macrophages in Fig. 5. Mitochondria form a highly dynamic network in the cells undergoing constant fusion and fission (Aravamudan et al. 2013). This dynamics is essential for mitochondrial function and maintenance and affects and interacts with important cell physiological processes like apoptosis, proliferation, oxidative stress, inflammation and others (Liesa et al. 2009; Tait and Green 2012). To estimate the balance between fusion and fission within the mitochondrial network, the connectivity of the network can be estimated in a 3D image stack using stereological investigation based on the Euler-Poincaré characteristic which describes the topological complexity of any structure (Gundersen et al. 1993). A structure is thereby defined by three topological phenomena: isolated islands I (or particles), bridges B (connections between particles) and holes H (enclosed cavities within islands). In mathematical terms the Euler-Poincaré characteristic (= Euler number) for any three-dimensional structures (*χ*_3_) can be expressed as follows:

*χ*_3_ = I – B + H.

In Fig. 5, two slices have been extracted out of the three-dimensional confocal microscopic dataset to illustrate the counting principle. Comparing these two images with each other allows counting new profiles of mitochondria which come into focus (white arrowheads). In addition, bridges can be identified and are defined as connections between particles which are present on the one section but not on the other section (red arrowheads). By acquiring an image stack of a whole cell, the connectivity of the entire mitochondrial network can be assessed.

Regarding light microscopy there have been impressive developments in 3D imaging, e.g. with the advent of multi-photon microscopy, light sheet microscopy or stimulated emission depletion (STED) microscopy. With these techniques it is possible to visualize larger volumes (e.g. up to a whole mouse lung) and/or achieve higher spatial resolution compared to confocal microscopy. Based on intrinsic fluorescence and specific staining, visualization of vessels and airways but also cell types in a larger context is possible. Hence, such 3D light microscopic imaging techniques offer the potential to count acini and alveoli but also cells based on the optical disector. Having the resolution and the larger accessible volume in mind these imaging techniques could combine some advantages of µCT and confocal microscopy. In general, with respect to application for design-based stereology it is advisable to validate new innovative three-dimensional imaging techniques with the current standard (which can be classical light microscopy). It has to be guaranteed that the structures of interest can be safely identified. In addition, confounders such as tissue deformation, tissue clearing effects and overprojection have to be controlled.

### Applications of 3D imaging-based stereology to the lung: X-ray based methods

Among the available three-dimensional imaging methods X-ray based imaging such as µCT has been used quite frequently in the last years to generate stereological data. In this context not only numbers or the Euler-Poincaré characteristic of defined anatomical structures were determined but also parameters which can easily be obtained from single two-dimensional sections following the principles of systematic uniform random sampling (Vasilescu et al. 2013, 2020). In 2013, Vasilescu et al. were the first to apply design-based stereology to three-dimensional µCT datasets following the American Thoracic Society / European Respiratory Society recommendations of quantitative morphology in the lung (Hsia et al. 2010) and compared data from µCT to data from classical histological sections (Vasilescu et al. 2013). The investigators used a multiresolution imaging approach and scanned whole mouse lungs at lower resolution. The low resolution datasets were used to determine the total volume of lung parenchyma according to the so-called Cavaleri principle. Based on systematic uniform random sampling, small volumes within lung parenchyma were then "virtually dissected" and scanned at high resolution, allowing identification of interalveolar septa and individual alveoli. These datasets were subjected to the determination of volume fractions of interalveolar septal walls and acinar airspaces but also the surface area of alveoli based on point and intersection counting. Two-dimensional µCT images were randomized within the volumetric datasets for this purpose. The “optical” disector principle was used to determine the number of alveoli. The investigators scrolled the sectional plane through the datasets and could integrate the information of the third dimension in the counting process. The same lungs were further processed for classical light microscopy and stereology to allow the direct comparison of the results between these two imaging techniques which demonstrated hardly any significant differences (Vasilescu et al. 2013). In this context it turned out that the strength of classical light microscopy is the higher resolution compared to µCT, and this can explain the finding that the surface area density of alveoli was significantly smaller in the µCT datasets compared to the light microscopic datasets. In this regard, the impact of the resolution of the applied imaging technique on the surface area data has been described decades ago comparing data obtained from TEM and classical light microscopic images (Gehr et al. 1978).

**Fig. 6:**
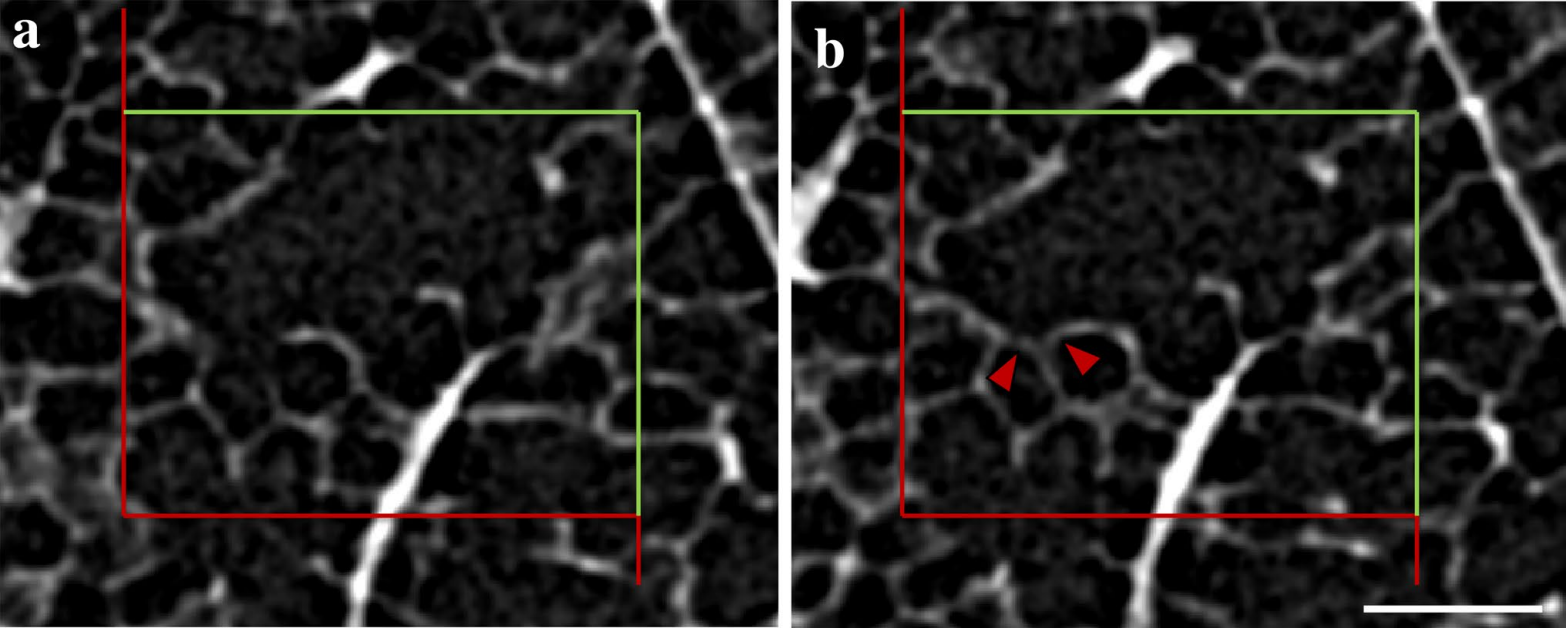
Counting of alveoli using µCT datasets. Two virtual slices taken from the µCT dataset in Fig. 2e are shown. The µCT allows scrolling through the imaged volume. In this dataset, the image in **a** is 4.5 µm above the one shown in **b**. An unbiased counting frame with a known area is projected on the slices. The counting frame has a “forbidden” line (red) and an acceptance line (green). While scrolling through the data set, counting events are defined by closure of an alveolus, provided that these closures are within the counting frame and do not touch the “forbidden” line. In **b** two alveoli are closed which are open in **a** (red arrowheads). Scale bar = 100 µm

The advantage of µCT compared to classical light microscopy is that it is non-destructive and allows the integration of structures within the three-dimensional context. With respect to stereological investigations this property is beneficial for the determination of the number of alveoli. By scrolling though the stack of images, which is comparable to the principle of the optical disector, a three-dimensional stereological probe is easily generated and alveoli which are open on the one section of the stack and closed at the next one (or the other way round) can efficiently be counted. Figure 6 shows two slices of a stack of µCT images and illustrates examples of the counting principle which is also based on the Euler-Poincaré characteristic of the network of alveolar openings: a connection (or bridge) between the edges of interalveolar septa protruding in the direction of the alveolar duct is counted (Ochs et al. 2004a; Hyde et al. 2004). For counting of alveoli according to stereological principles, volumetric µCT datasets have been used by several investigators within recent years. McDonough and co-workers investigated regional differences within the human lung. The regional density of alveoli correlated with lung height and the largest density of alveoli was found in the apex and the lowest in the base of the lung. The volume fraction of ductal airspaces, however, behaved the other way round (McDonough et al. 2015). Following a systematic uniform random sampling, Vasilescu et al. (2020) extensively investigated air-filled human donor lungs at a defined end-expiratory airway opening pressure using design-based stereology for volumetric µCT datasets and compared these data to data published before including also papers presenting data obtained from light and electron microcopy (Vasilescu et al. 2020). Aside from classical stereological parameters such as the volumes of alveolar and ductal airspaces, volume and thickness of interalveolar septa or the surface area of alveoli available for gas exchange, the investigators also determined the number of alveoli and the number of acini per lung using the disector.

While the determination of the number of alveoli has been done before in an unbiased manner using classical light microcopy and stereology in different species (Hyde et al. 2004, 2007; Ochs et al. 2004a; Tschanz et al. 2014b) there is only one publication introducing a design-based stereological method for histological sections to determine the number of ventilatory units in mice (= acini) (Wulfsohn et al. 2010). This method is based on a physical disector to determine the Euler-Poincaré characteristic of the conducting airways which end in mice (and other rodents) at the bronchiole-alveolar duct junctions. In mice this is a clearly defined anatomical structure where the cuboidal epithelium of bronchioles abruptly turns into the squamous epithelium of the alveoli. The alveolar ducts follow the course of the bronchioles and can be considered as an airway surrounded by a sleeve of alveoli. In mice, the number of these junctions is equivalent to the number of acini, the functional unit of the lung (Wulfsohn et al. 2010). In humans the terminal bronchioles (= the last purely conductive generation of the airway tree) do not directly branch into the alveolar ducts. Instead there are around three branching generations of respiratory bronchioles in which the respiratory epithelium is interrupted by protuberances of alveoli lined by squamous epithelium. Since the acinus is defined as the lung parenchyma distal to the terminal bronchioles (Haefeli-Bleuer and Weibel 1988; Rodriguez et al. 1987) its entrance cannot be safely identified using pairs of two-dimensional light microscopic sections for a physical disector. Hence, the use of volumetric µCT datasets made the determination of the numbers of terminal bronchioles possible in those lungs which have respiratory bronchioles. Three-dimensional information allowed clear identification of the first generation of respiratory bronchioles based on the focal occurrence of alveoli e.g. by scrolling along the airway. Hence, the related “mother airway” corresponded to the terminal bronchiole. The identification and unbiased sampling of the terminal bronchioles following the “optical” disector principle also allowed to perform further (non-stereological) measurements such as determination of the length and wall thickness of the terminal bronchioles or counting of alveolar attachments along the terminal bronchioles (Vasilescu et al. 2020). The number of terminal bronchioles and related morphometrical data has been shown to be of relevance for lung disease such as chronic obstructive pulmonary disease (COPD) (McDonough et al. 2011; Vasilescu et al. 2019), idiopathic pulmonary fibrosis (Verleden et al. 2020) or cystic fibrosis (Boon et al. 2016).

**Fig. 7:**
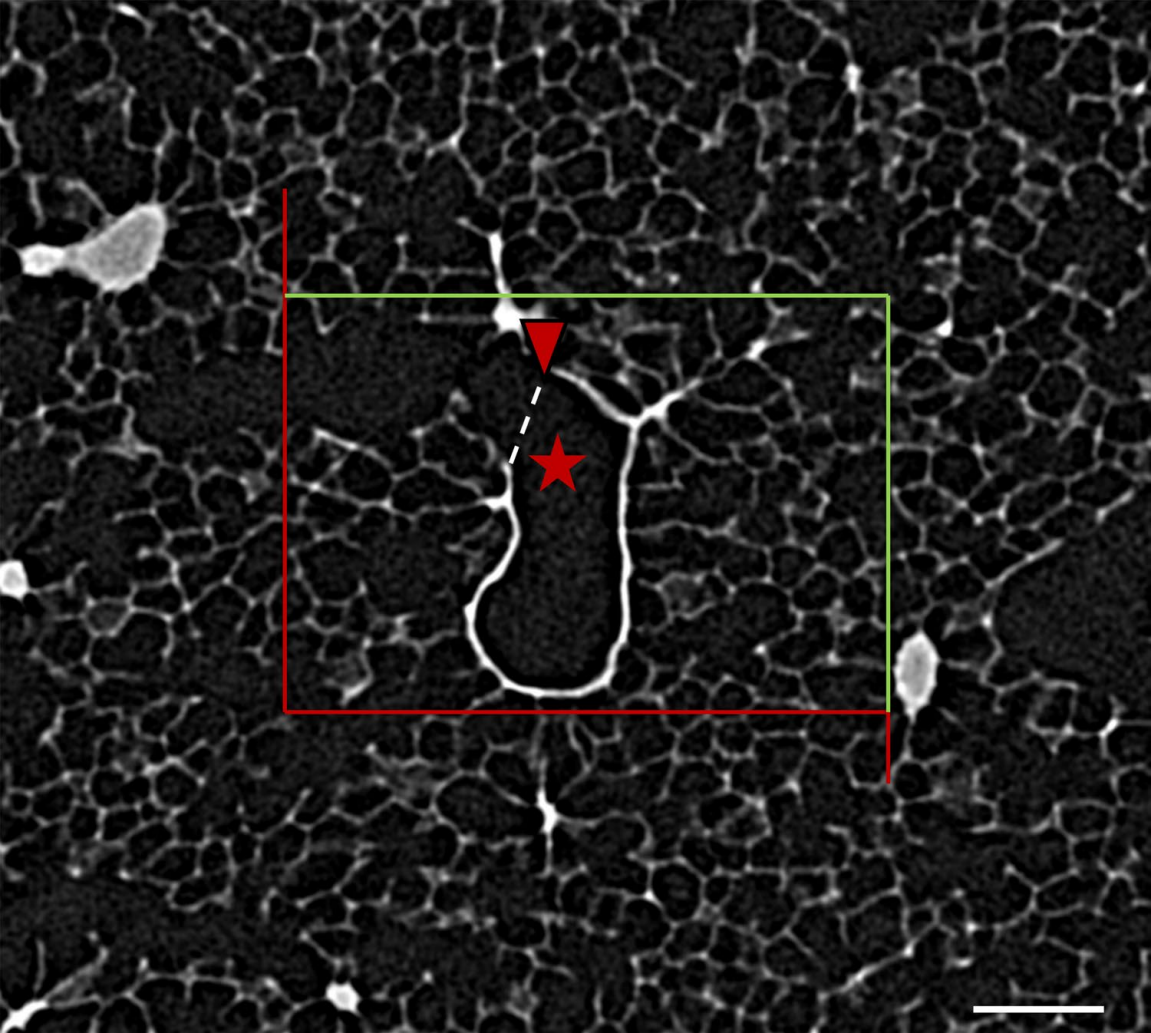
Entrance into an acinus. µCT image showing the bronchiole-alveolar duct junction based on the appearance of the airway wall terminal bronchiole which shows an abrupt decrease in thickness (red arrow) when entering the alveolated acinus. The white dashed line indicates the plane where the terminal bronchiole (asterisk) enters the acinus. µCT datasets were used to count these junctions in a defined volume of lung parenchyma by scrolling through the stack according to the “optical” disector principle (for details, see Barré et al. 2014; McDonough et al. 2011). Scale bar = 200 µm

Three-dimensional imaging of the lung not only gave important insights into the pathophysiology of human lung diseases but also into postnatal lung development (Schittny 2018). High resolution, synchrotron radiation-based X-ray tomography has been shown to be useful to identify the bronchiole-alveolar duct junction in rat lungs allowing segmentation of complete acini and investigation of the internal acinar structure including the branching pattern of intra-acinar pathways (Haberthür et al. 2013). Similar to the µCT datasets from human lungs (McDonough et al. 2011) it is possible to scroll through the virtual (nearly two-dimensional) slices of the three-dimensional dataset. The bronchiole-alveolar duct junctions in rat lungs were identified based on the change in the thickness of the wall which indicates the functional change of the purely convective conducting airways (thick walls) to the gas exchange region, the acinus (thin wall) (Barré et al. 2014). Figure 7 illustrates an example of the bronchiole-alveolar duct junction in a mouse lung. Investigating different ages after birth, Barré et al. could demonstrate that the number of acini remains roughly stable during postnatal development ranging from age 4 to 60 days so that it can be concluded that all acini are already formed before the age of 4 days during the saccular stage (Barré et al. 2016). Postnatal lung growth is characterized by an increase in the sizes of the acini but not their numbers per lung.

### The Euler-Poincaré characteristic: a relevant parameter in lung research

As mentioned above, the Euler-Poincaré characteristic was introduced in lung stereology for estimating the number of alveoli. Due to their openings into alveolar ducts, alveoli are connected and incomplete "particles" which could therefore not be counted in physical disectors in the ordinary way. Twenty years after the development of the disector principle (Sterio 1984), this seemingly disadvantageous property was actually used to solve this long-standing problem in lung biology. Based on the topological properties of the network of alveolar openings, the Euler number of this network was estimated using physical disectors in classical light microscopy. Thus, alveolar openings eventually became the counting event for disector-based estimation of alveolar number (Ochs et al. 2004a; Hyde et al. 2004).

The Euler-Poincaré characteristic has also been applied in biomedical research of the lung and other organs to quantify the connectivity of capillaries using the classical physical disector method based on consecutive light microscopic sections (Willführ et al. 2015; Nyengaard and Marcussen 1993). In the lung, Willführ et al. quantified the number of capillary loops within interalveolar septa during postnatal lung development and could demonstrate that it increased from 0.106 billion on day 6 to 1.030 billion on day 42 after birth in rats. Although these data were generated based on two-dimensional light microscopy combined with the physical disector, the counting rules could also be applied to registered three-dimensional datasets based on SBF-SEM or SEM-based array tomography using the “optical” disector principle (Mühlfeld et al. 2018).

Other investigators used the Euler-Poincaré characteristic to characterize the complexity of the fibroblast foci in human idiopathic pulmonary fibrosis (IPF) (Cool et al. 2006; Jones et al. 2016). The fibroblast foci are the histopathological hallmark of IPF and the volume fraction of fibroblast foci within lung parenchyma has been shown to correlate with disease progression and survival (Knudsen et al. 2017). Cool and co-authors generated serial microscopic sections of surgical lung biopsies from patients with IPF and applied the physical disector for counting of islands as well as bridges between fibroblast foci to estimate the connectivity (Cool et al. 2006). Ten years later, Jones et al. applied µCT which they correlated to classical histological sections to identify fibroblast foci and determined the Euler-Poincaré characteristic based on the three-dimensional µCT datasets combined with the “optical” disector principle (Jones et al. 2016). Both studies described the fibroblast foci as morphologically very complex structures with variability in shape and volume. However, while Cool et al. described rather a reticulum of fibroblast foci with a quite high degree of connectivity, Jones et al. found hardly any bridges between fibroblast foci and came to the conclusion that there is no evidence for interconnectivity so that these fibroblast foci are rather isolated particles than a reticulum. The different findings described by these studies might result from the different resolutions (light microscopy vs. µCT) and varying definitions in the counting events. Cool et al. stained light microscopic serial sections for collagen and this collagen staining guided the counting of islands and bridges in the physical disector (Cool et al. 2006), while Jones et al. used µCT which has a lower resolution and does not allow the use of stains to differentiate between the histological components for the stereological quantification of the Euler-Poincaré characteristic (Jones et al. 2016).

### Summary and outlook

Taken together, volumetric imaging has definitely improved the efficiency in determining stereological parameters of complex anatomical structures in the lung (Table 2). In addition, three-dimensional imaging techniques might also provide new opportunities to perform lung stereology in an easy and unbiased way in the future. This potential may be illustrated by one example: In 1985 Gundersen and Jensen introduced the point-sampled intercept lengths method to determine the so-called volume-weighted mean volume (or star volume) of arbitrary anatomical structures (Gundersen and Jensen 1985). This parameter is very attractive since it is easy to implement and can be applied to any complex structure independent from assumptions regarding shape, curvature or spatial orientation. The method is based on test points which are randomly superimposed on the tissue under study. Those structures hit by the test points are measured: from each test point straight lines radiate in a systematic random direction through the structure of interest so that length measurements from border to border of the sampled structure can be performed. This method has so far been performed on two-dimensional sections to determine the star volume of the trabecular bone and bone marrow cavities (Vesterby et al. 1989) as well as the intervillous space of the placenta (Mayhew and Wadrop 1994). The name star volume has been derived from the fact that more than one straight line radiates from the sampling point to the borders of the structure of interest so that more than one length measurement is performed: the point and radiating test lines look like a star. For anisotropic structures it is, however, essential that the directions of these lines are random in three dimensions. For calculation of the star volume (V(star)) of the sampled structure the mean of the third power of length measurements (l^3^) is used in the following formula:

**Table 2:** 3D imaging techniques and their application for design-based stereology in lung research

3D imaging technique	Visualized lung structure	Quantitative parameter	Stereological method	Strengths/limitations	Use recommended?	Reference
SBF-SEM	Alveolar epithelial type II cells; Lamellar bodies	Number of alveolar epithelial cells; Number of lamellar bodies	Disector	Efficiency compared to TEM combined with physical disector more than doubtful	No	None
	Alveolar capillary network	Connectivity of capillary network; Number of capillary loops	Disector	Clear identification of counting event requires 3D dataset	Yes	Buchacker et al. 2019; Mühlfeld et al. 2018
Confocal microscopy	Alveolar epithelial type II cells	Number of alveolar epithelial type II cells	Optical disector	Higher efficiency compared to physical disector; Requires specific staining	Yes	Jansing et al. 2018
	Alveolar and interstitial macrophages	Number of alveolar and interstitial macrophages			Yes	Hume et al. 2020
µCT (human)	Conducting airways	Number of terminal and respiratory bronchioles	Disector	Clear identification of counting event requires 3D dataset	Yes	McDonough et al. 2011; Vasilescu et al. 2020
µCT (mice or rats)	Conducting airways and BADJ	Number of ventilatory units / acini	Disector	Higher efficiency compared to physical disector based on 2D images	Yes	Barré et al. 2014; Barré et al. 2016
µCT (mice and humans)	Acinus	Number-weighted mean volume of acinus	Cavalieri method	Identification of complete acinus requires 3D datasets	Yes	None
	Alveoli, interalveolar septa	Number of alveoli; Surface area of alveoli; Mean linear intercept length of distal airspaces; Volume composition of lung parenchyma	Disector; Point and intersection counting	Higher efficiency compared to physical disector based on 2D images	Yes	Vasilescu et al. 2012a; McDonough et al. 2015; Vasilescu et al. 2020

BADJ = bronchiole-alveolar duct junction

$$V(star)=\left(\frac{\pi }{3}\right){l}^{3}$$

**Fig. 8:**
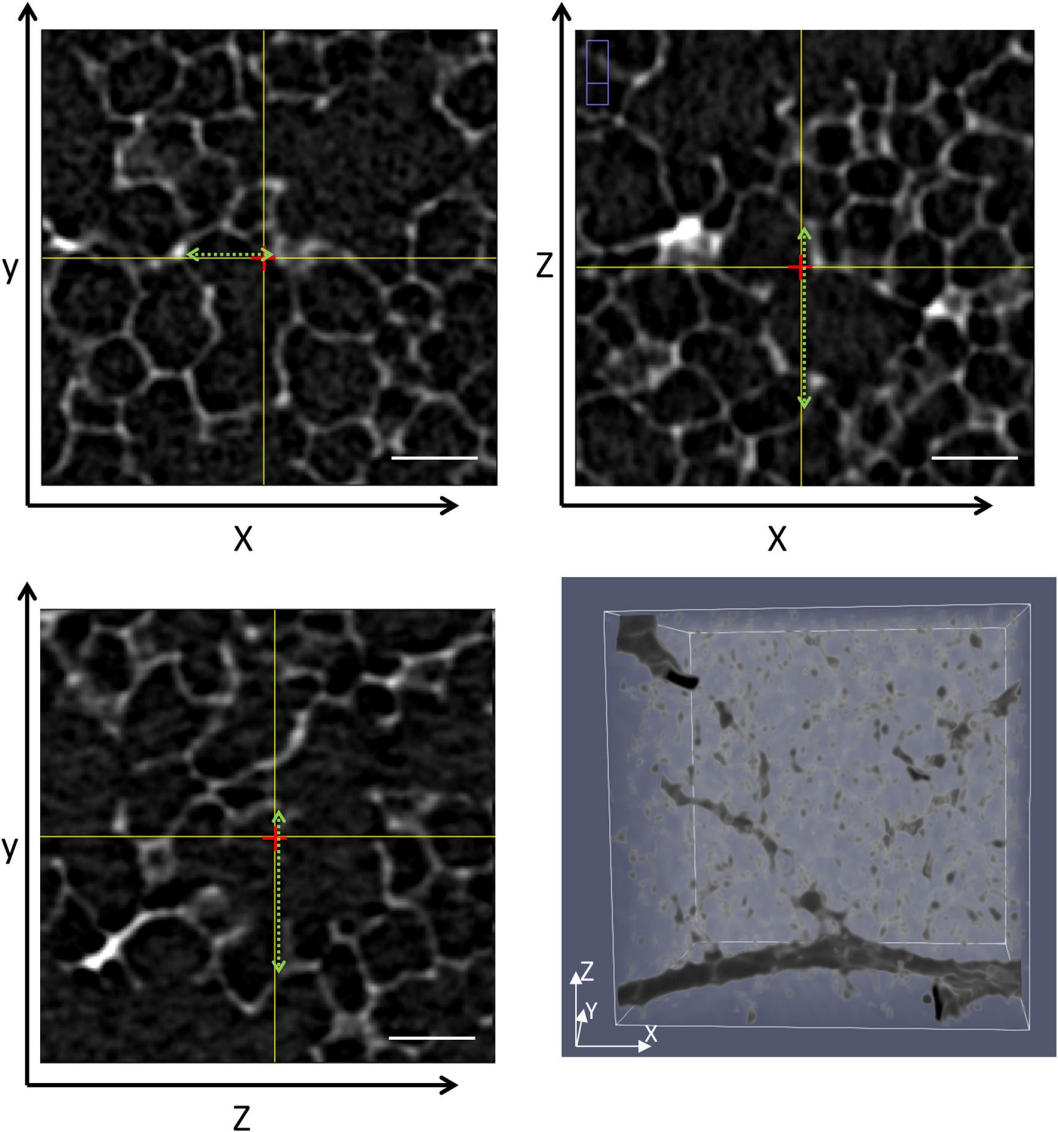
Star volume. A virtual tissue block has been randomly taken from the µCT dataset illustrated in Fig. 2e. This cube has an edge length of 300 pixel which corresponds to 450 µm. A random pixel (labeled with a red cross) was taken within this coordinate system using ImageJ, and orthogonal slices, representing all planes in three-dimensional space were generated. The locations of the orthogonal slices on each plane are indicated by the yellow lines. Length measurements along the three axes traversing trough the test point (red) were performed from wall to wall of the acinar airspace (scattered green line). These length measurements can be used to estimate star volume as described in the text. Scale bar: 80 µm

In the lung, star volume could be applied to the acinar airspaces in three-dimensional datasets by positioning a spatial point grid in a uniform random manner in the dataset for sampling acinar airspaces. Straight test lines which radiate from the test point and reflect all three dimensions in space can be used to measure lengths from wall to wall so that star volume of acinar airspaces can be calculated according to the above mentioned formula (Fig. 8). Having the physiology of gas transport in mind this parameter appears to be of high functional relevance since oxygen is transported within the acinar airspaces primarily by diffusion. The star volume of the acinar airspace would, in this context, provide a physiologically relevant parameter since it describes a volume through which oxygen needs to diffuse to reach the surface area of the acinus where the blood-gas barrier is located (Sapoval et al. 2002). Moreover, the distribution of length measurements from a test point contains information about individual diffusion pathway lengths for a given oxygen molecule at a given position within the acinus. In lung disease such as pulmonary emphysema star volume of acinar airspaces might be a very sensitive parameter since length measurements of acinar airspaces are raised to the third power.

In summary, the symbiosis of design-based stereology and three-dimensional imaging techniques allows for quantitative 3D lung imaging at virtually all scales and resolutions. This provides the methodological basis for comprehensive morphomics (Ferguson et al. 2017; Mayhew and Lucocq 2015; Lucocq et al. 2015) of the lung under physiological and pathological conditions. Stereology is deeply rooted in stochastic geometry and can be used as a toolbox for sampling and measuring biological objects in microscopy. As such, it is independent from particular imaging techniques but universally applicable. Any new development in microscopy broadens the potential applications of stereology. Therefore, 3D imaging techniques do not make stereology dispensable. On the contrary, they offer unprecedented opportunities for smart applications of stereology in the future.

## References

[CR1] Albert K, Krischer J-M, Pfaffenroth A, Wilde S, Lopez-Rodriguez E, Braun A, Smith BJ, Knudsen L (2020). Hidden microatelectases increase vulnerability to ventilation-induced lung injury. Front Physiol.

[CR2] Aravamudan B, Thompson MA, Pabelick CM, Prakash YS (2013). Mitochondria in lung diseases. Expert Rev Respir Med.

[CR3] Baddeley A, Vedel Jensen EB (2005). Stereology for statisticians.

[CR4] Barré SF, Haberthür D, Cremona TP, Stampanoni M, Schittny JC (2016). The total number of acini remains constant throughout postnatal rat lung development. Am J Physiol Lung Cell Mol Physiol.

[CR5] Barré SF, Haberthür D, Stampanoni M, Schittny JC (2014) Efficient estimation of the total number of acini in adult rat lung. Physiol Rep 2 (7). doi:10.14814/phy2.12063 10.14814/phy2.12063 PMC4187566 24997068

[CR6] Beers MF, Knudsen L, Tomer Y, Maronn J, Zhao M, Ochs M, Mulugeta S (2017). Aberrant lung remodeling in a mouse model of surfactant dysregulation induced by modulation of the Abca3 gene. Ann Anat.

[CR7] Beike L, Wrede C, Hegermann J, Lopez-Rodriguez E, Kloth C, Gauldie J, Kolb M, Maus UA, Ochs M, Knudsen L (2019). Surfactant dysfunction and alveolar collapse are linked with fibrotic septal wall remodeling in the TGF-β1-induced mouse model of pulmonary fibrosis. Lab Invest.

[CR8] Bjugn R, Gundersen HJ (1993). Estimate of the total number of neurons and glial and endothelial cells in the rat spinal cord by means of the optical disector. J Comp Neurol.

[CR9] Boon M, Verleden SE, Bosch B, Lammertyn EJ, McDonough JE, Mai C, Verschakelen J, Kemner-van de Corput M, Tiddens HA, Proesmans M, Vermeulen FL, Verbeken EK, Cooper J, Van Raemdonck DE, Decramer M, Verleden GM, Hogg JC, Dupont LJ, Vanaudenaerde BM, De Boeck K (2016). Morphometric Analysis of Explant Lungs in Cystic Fibrosis. Am J Respir Crit Care Med.

[CR10] Boyce R, Ebert D, Youngs T, Paddock C, Mosekilde L, Stevens M, Gundersen H (1995). Unbiased estimation of vertebral trabecular connectivity in calcium-restricted ovariectomized minipigs. Bone.

[CR11] Brandenberger C, Ochs M, Mühlfeld C (2015). Assessing particle and fiber toxicology in the respiratory system: the stereology toolbox. Part Fibre Toxicol.

[CR12] Buchacker T, Mühlfeld C, Wrede C, Wagner WL, Beare R, McCormick M, Grothausmann R (2019). Assessment of the Alveolar Capillary Network in the Postnatal Mouse Lung in 3D Using Serial Block-Face Scanning Electron Microscopy. Front Physiol.

[CR13] Cool CD, Groshong SD, Rai PR, Henson PM, Stewart JS, Brown KK (2006). Fibroblast foci are not discrete sites of lung injury or repair: the fibroblast reticulum. Am J Respir Crit Care Med.

[CR14] Cruz-Orive LM, Weibel ER (1981). Sampling designs for stereology. J Microsc.

[CR15] Dorph-Petersen K, Nyengaard JR, Gundersen HJ (2001). Tissue shrinkage and unbiased stereological estimation of particle number and size. J Microsc.

[CR16] Elias H (1949). The liver cord concept after one hundred years. Science.

[CR17] Elias H (1971). Three-dimensional structure identified from single sections. Science.

[CR18] Elias H, Hennig A, Schwartz DE (1971). Stereology: applications to biomedical research. Physiol Rev.

[CR19] Elias H (1972). Die Lunge.

[CR20] Elias H, Pauly JE, Burns ER (1978). Histology and Human Microanatomy.

[CR21] Fehrenbach H, Wahlers T, Ochs M, Brasch F, Schmiedl A, Hirt S, Haverich A, Richter J (1998) Ultrastructural pathology of the alveolar type II pneumocytes of human donor lungs. Electron microscopy, stereology, and microanalysis. Virchows Arch 432 (3):229–239. 10.1007/s004280050160 9532002

[CR22] Ferguson S, Steyer AM, Mayhew TM, Schwab Y, Lucocq JM (2017). Quantifying Golgi structure using EM: combining volume-SEM and stereology for higher throughput. Histochem Cell Biol.

[CR23] Fung YC, Sobin SS (1969). Theory of sheet flow in lung alveoli. J Appl Physiol.

[CR24] Funke M, Knudsen L, Lagares D, Ebener S, Probst CK, Fontaine BA, Franklin A, Kellner M, Kuhnel M, Matthieu S, Grothausmann R, Chun J, Roberts JD, Ochs M, Tager AM (2016). Lysophosphatidic Acid Signaling through the Lysophosphatidic Acid-1 Receptor Is Required for Alveolarization. Am J Respir Cell Mol Biol.

[CR25] Gehr P, Bachofen M, Weibel ER (1978). The normal human lung: ultrastructure and morphometric estimation of diffusion capacity. Respir Physiol.

[CR26] Gokhale AM, Evans RA, Mackes JL, Mouton PR (2004). Design-based estimation of surface area in thick tissue sections of arbitrary orientation using virtual cycloids. J Microsc.

[CR27] Grothausmann R, Knudsen L, Ochs M, Muhlfeld C (2017). Digital 3D reconstructions using histological serial sections of lung tissue including the alveolar capillary network. Am J Physiol Lung Cell Mol Physiol.

[CR28] Gundersen HJ (1977). Notes on the estimation of the numerical density of arbitrary profiles: the edge effect. J Microsc.

[CR29] Gundersen HJ (1986) Stereology of arbitrary particles. A review of unbiased number and size estimators and the presentation of some new ones, in memory of William R. Thompson. J Microsc 143 (Pt 1):3–45. 3761363

[CR30] Gundersen HJ (1988). The nucleator. J Microsc.

[CR31] Gundersen HJ (2002). The smooth fractionator. J Microsc.

[CR32] Gundersen H, Osterby R (1981). Optimizing sampling efficiency of stereological studies in biology: or 'do more less well!'. J Microsc.

[CR33] Gundersen HJ, Bagger P, Bendtsen T, Evans S, Korbo L, Marcussen N, Møller A, Nielsen K, Nyengaard JR, Pakkenberg B (1988). The new stereological tools: disector, fractionator, nucleator and point sampled intercepts and their use in pathological research and diagnosis. APMIS.

[CR34] Gundersen HJ, Jensen E (1985). Stereological estimation of the volume-weighted mean volume of arbitrary particles observed on random sections. J Microsc.

[CR35] Gundersen HJ, Jensen E (1987). The efficiency of systematic sampling in stereology and its prediction. J Microsc.

[CR36] Gundersen HJ, Boyce RW, Nyengaard JR, Odgaard A (1993). The Conneulor: unbiased estimation of connectivity using physical disectors under projection. Bone.

[CR37] Haberthür D, Barré SF, Tschanz SA, Yao E, Stampanoni M, Schittny JC (2013). Visualization and stereological characterization of individual rat lung acini by high-resolution X-ray tomographic microscopy. J Appl Physiol.

[CR38] Haefeli-Bleuer B, Weibel E (1988). Morphometry of the human pulmonary acinus. Anat Rec.

[CR39] Hildebrandt S (2012). The anatomist Hans Elias: a Jewish German in exile. Clin Anat.

[CR40] Howard CV, Reed MG (2005). Unbiased stereology.

[CR41] Hsia CC, Hyde DM, Ochs M, Weibel ER (2010). An official research policy statement of the American Thoracic Society/European Respiratory Society: standards for quantitative assessment of lung structure. Am J Respir Crit Care Med.

[CR42] Hume PS, Gibbings SL, Jakubzick CV, Tuder RM, Curran-Everett D, Henson PM, Smith BJ, Janssen WJ (2020). Localization of Macrophages in the Human Lung via Design-based Stereology. Am J Respir Crit Care Med.

[CR43] Hyde DM, Tyler N, Putney L, Singh P, Gundersen H (2004). Total number and mean size of alveoli in mammalian lung estimated using fractionator sampling and unbiased estimates of the Euler characteristic of alveolar openings. Anat Rec A Discov Mol Cell Evol Biol.

[CR44] Hyde DM, Blozis SA, Avdalovic MV, Putney LF, Dettorre R, Quesenberry NJ, Singh P, Tyler NK (2007). Alveoli increase in number but not size from birth to adulthood in rhesus monkeys. Am J Physiol Lung Cell Mol Physiol.

[CR45] Jansing NL, Patel N, McClendon J, Redente EF, Henson PM, Tuder RM, Hyde DM, Nyengaard JR, Zemans RL (2018). Flow Cytometry Underestimates and Planimetry Overestimates Alveolar Epithelial Type 2 Cell Expansion after Lung Injury. Am J Respir Crit Care Med.

[CR46] Jones MG, Fabre A, Schneider P, Cinetto F, Sgalla G, Mavrogordato M, Jogai S, Alzetani A, Marshall BG, O'Reilly KM, Warner JA, Lackie PM, Davies DE, Hansell DM, Nicholson AG, Sinclair I, Brown KK, Richeldi L (2016). Three-dimensional characterization of fibroblast foci in idiopathic pulmonary fibrosis. JCI Insight.

[CR47] Jung A, Allen L, Nyengaard J, Gundersen H, Richter J, Hawgood S, Ochs M (2005). Design-based stereological analysis of the lung parenchymal architecture and alveolar type II cells in surfactant protein A and D double deficient mice. Anat Rec A Discov Mol Cell Evol Biol.

[CR48] Kellner M, Heidrich M, Beigel R, Lorbeer RA, Knudsen L, Ripken T, Heisterkamp A, Meyer H, Kühnel MP, Ochs M (2012). Imaging of the mouse lung with scanning laser optical tomography (SLOT). J Appl Physiol.

[CR49] Kellner M, Wehling J, Warnecke G, Heidrich M, Izykowski N, Vogel-Claussen J, Lorbeer RA, Antonopoulos G, Janciauskiene S, Grothausmann R, Knudsen L, Ripken T, Meyer H, Kreipe H, Ochs M, Jonigk D, Kuhnel MP (2015). Correlating 3D morphology with molecular pathology: fibrotic remodelling in human lung biopsies. Thorax.

[CR50] Knudsen L, Ruppert C, Ochs M (2017). Tissue remodelling in pulmonary fibrosis. Cell Tissue Res.

[CR51] Knudsen L, Waizy H, Fehrenbach H, Richter J, Wahlers T, Wittwer T, Ochs M (2011). Ultrastructural changes of the intracellular surfactant pool in a rat model of lung transplantation-related events. Respir Res.

[CR52] Knust J, Ochs M, Gundersen H, Nyengaard J (2009). Stereological estimates of alveolar number and size and capillary length and surface area in mice lungs. Anat Rec.

[CR53] Kroustrup J, Gundersen H (2001). Estimating the number of complex particles using the ConnEulor principle. J Microsc.

[CR54] Kubínová L, Janácek J (1998). Estimating surface area by the isotropic fakir method from thick slices cut in an arbitrary direction. J Microsc.

[CR55] Kubínová L, Janáček J (2015). Confocal stereology: an efficient tool for measurement of microscopic structures. Cell Tissue Res.

[CR56] Liesa M, Palacín M, Zorzano A (2009). Mitochondrial dynamics in mammalian health and disease. Physiol Rev.

[CR57] Lucocq JM, Mayhew TM, Schwab Y, Steyer AM, Hacker C (2015). Systems biology in 3D space–enter the morphome. Trends Cell Biol.

[CR58] Lutz D, Gazdhar A, Lopez-Rodriguez E, Ruppert C, Mahavadi P, Gunther A, Klepetko W, Bates JH, Smith B, Geiser T, Ochs M, Knudsen L (2015). Alveolar Derecruitment and Collapse Induration as Crucial Mechanisms in Lung Injury and Fibrosis. Am J Respir Cell Mol Biol.

[CR59] Mattfeldt T, Mall G, Gharehbaghi H, Möller P (1990). Estimation of surface area and length with the orientator. J Microsc.

[CR60] Mayhew TM, Lucocq JM (2015). From gross anatomy to the nanomorphome: stereological tools provide a paradigm for advancing research in quantitative morphomics. J Anat.

[CR61] Mayhew TM, Wadrop E (1994). Placental morphogenesis and the star volumes of villous trees and intervillous pores. Placenta.

[CR62] McDonough JE, Knudsen L, Wright AC, Elliott WM, Ochs M, Hogg JC (2015). Regional differences in alveolar density in the human lung are related to lung height. J Appl Physiol.

[CR63] McDonough JE, Yuan R, Suzuki M, Seyednejad N, Elliott WM, Sanchez PG, Wright AC, Gefter WB, Litzky L, Coxson HO, Paré PD, Sin DD, Pierce RA, Woods JC, McWilliams AM, Mayo JR, Lam SC, Cooper JD, Hogg JC (2011). Small-airway obstruction and emphysema in chronic obstructive pulmonary disease. N Engl J Med.

[CR64] Møller A, Strange P, Gundersen H (1990). Efficient estimation of cell volume and number using the nucleator and the disector. J Microsc.

[CR65] Mühlfeld C, Hegermann J, Wrede C, Ochs M (2015). A review of recent developments and applications of morphometry/stereology in lung research. Am J Physiol Lung Cell Mol Physiol.

[CR66] Mühlfeld C, Ochs M (2013) Quantitative microscopy of the lung: a problem-based approach. Part 2: stereological parameters and study designs in various diseases of the respiratory tract. Am J Physiol Lung Cell Mol Physiol 305 (3):L205–221. doi:10.1152/ajplung.00427.2012 10.1152/ajplung.00427.2012 23709622

[CR67] Mühlfeld C, Weibel ER, Hahn U, Kummer W, Nyengaard JR, Ochs M (2010). Is length an appropriate estimator to characterize pulmonary alveolar capillaries? A critical evaluation in the human lung. Anat Rec.

[CR68] Mühlfeld C, Wrede C, Knudsen L, Buchacker T, Ochs M, Grothausmann R (2018). Recent developments in 3D reconstruction and stereology to study the pulmonary vasculature. Am J Physiol Lung Cell Mol Physiol.

[CR69] Nyengaard JR, Gundersen HJ (1992). The isector: a simple and direct method for generating isotropic, uniform random sections from small specimens. J Microsc.

[CR70] Nyengaard JR, Gundersen HJ (2006). Sampling for stereology in lungs. Eur Respir Rev.

[CR71] Nyengaard JR, Marcussen N (1993). The number of glomerular capillaries estimated by an unbiased and efficient stereological method. J Microsc.

[CR72] Ochs M, Knudsen L, Hegermann J, Wrede C, Grothausmann R, Mühlfeld C (2016). Using electron microscopes to look into the lung. Histochem Cell Biol.

[CR73] Ochs M, Nyengaard LR, Jung A, Knudsen L, Voigt M, Wahlers T, Richter J, Gundersen HJG (2004). The number of alveoli in the human lung. Am J Respir Criti Care Med.

[CR74] Ochs M, Knudsen L, Allen L, Stumbaugh A, Levitt S, Nyengaard JR, Hawgood S (2004). GM-CSF mediates alveolar epithelial type II cell changes, but not emphysema-like pathology, in SP-D deficient mice. Am J Physiol Lung Cell Mol Physiol.

[CR75] Ochs M (2020). And then I met Ewald Weibel. Am J Physiol Lung Cell Mol Physiol.

[CR76] Rasmusson A, Hahn U, Larsen JO, Gundersen HJG, Vedel Jensen EB, Nyengaard JR (2013). The spatial rotator. J Microsc.

[CR77] Rodriguez M, Bur S, Favre A, Weibel ER (1987). Pulmonary acinus: geometry and morphometry of the peripheral airway system in rat and rabbit. Am J Anat.

[CR78] Sapoval B, Filoche M, Weibel ER (2002). Smaller is better–but not too small: a physical scale for the design of the mammalian pulmonary acinus. Proc Natl Acad Sci U S A.

[CR79] Schipke J, Banmann E, Nikam S, Voswinckel R, Kohlstedt K, Loot AE, Fleming I, Mühlfeld C (2014). The number of cardiac myocytes in the hypertrophic and hypotrophic left ventricle of the obese and calorie-restricted mouse heart. J Anat.

[CR80] Schittny JC (2018). How high resolution 3-dimensional imaging changes our understanding of postnatal lung development. Histochem Cell Biol.

[CR81] Schneider JP, Ochs M (2014). Alterations of mouse lung tissue dimensions during processing for morphometry: a comparison of methods. Am J Physiol Lung Cell Mol Physiol.

[CR82] Schneider JP, Wrede C, Hegermann J, Weibel ER, Mühlfeld C, Ochs M (2019). On the Topological Complexity of Human Alveolar Epithelial Type 1 Cells. Am J Respir Crit Care Med.

[CR83] Schneider JP, Hegermann J, Wrede C (2020). Volume electron microscopy: analyzing the lung. Histochem Cell Biol (in press).

[CR84] Sterio D (1984). The unbiased estimation of number and sizes of arbitrary particles using the disector. J Microsc.

[CR85] Tait SW, Green DR (2012). Mitochondria and cell signalling. J Cell Sci.

[CR86] Tandrup T, Gundersen HJ, Jensen EB (1997). The optical rotator. J Microsc.

[CR87] Tschanz S, Schneider JP, Knudsen L (2014). Design-based stereology: Planning, volumetry and sampling are crucial steps for a successful study. Ann Anat.

[CR88] Tschanz SA, Salm LA, Roth-Kleiner M, Barré SF, Burri PH, Schittny JC (2014). Rat lungs show a biphasic formation of new alveoli during postnatal development. J Appl Physiol.

[CR89] Vanhecke D, Studer D, Ochs M (2007). Stereology meets electron tomography: towards quantitative 3D electron microscopy. J Struct Biol.

[CR90] Vasilescu DM, Gao Z, Saha PK, Yin L, Wang G, Haefeli-Bleuer B, Ochs M, Weibel ER, Hoffman EA (2012). Assessment of morphometry of pulmonary acini in mouse lungs by nondestructive imaging using multiscale microcomputed tomography. Proc Natl Acad Sci U S A.

[CR91] Vasilescu DM, Klinge C, Knudsen L, Yin LL, Wang G, Weibel ER, Ochs M, Hoffman EA (2013). Stereological assessment of mouse lung parenchyma via nondestructive, multiscale micro-CT imaging validated by light microscopic histology. J Appl Physiol.

[CR92] Vasilescu DM, Knudsen L, Ochs M, Weibel ER, Hoffman EA (2012). Optimized murine lung preparation for detailed structural evaluation via micro-computed tomography. J Appl Physiol.

[CR93] Vasilescu DM, Martinez FJ, Marchetti N, Galbán CJ, Hatt C, Meldrum CA, Dass C, Tanabe N, Reddy RM, Lagstein A, Ross BD, Labaki WW, Murray S, Meng X, Curtis JL, Hackett TL, Kazerooni EA, Criner GJ, Hogg JC, Han MK (2019). Noninvasive Imaging Biomarker Identifies Small Airway Damage in Severe Chronic Obstructive Pulmonary Disease. Am J Respir Crit Care Med.

[CR94] Vasilescu DM, Phillion AB, Kinose D, Verleden SE, Vanaudenaerde BM, Verleden GM, Van Raemdonck D, Stevenson CS, Hague CJ, Han MK, Cooper JD, Hackett TL, Hogg JC (2020). Comprehensive stereological assessment of the human lung using multiresolution computed tomography. J Appl Physiol.

[CR95] Verleden SE, Tanabe N, McDonough JE, Vasilescu DM, Xu F, Wuyts WA, Piloni D, De Sadeleer L, Willems S, Mai C, Hostens J, Cooper JD, Verbeken EK, Verschakelen J, Galban CJ, Van Raemdonck DE, Colby TV, Decramer M, Verleden GM, Kaminski N, Hackett TL, Vanaudenaerde BM, Hogg JC (2020). Small airways pathology in idiopathic pulmonary fibrosis: a retrospective cohort study. Lancet Respir Med.

[CR96] Vesterby A, Gundersen HJ, Melsen F (1989). Star volume of marrow space and trabeculae of the first lumbar vertebra: sampling efficiency and biological variation. Bone.

[CR97] Weibel ER (1963). Morphometry of the human lung.

[CR98] Weibel ER, Federspiel W, Fryder-Doffey F, Hsia C, König M, Stalder-Navarro V, Vock R (1993). Morphometric model for pulmonary diffusing capacity. I Membrane diffusing capacity Respir Physiol.

[CR99] Weibel ER, Hsia CC, Ochs M (2007). How much is there really? Why stereology is essential in lung morphometry. J Appl Physiol.

[CR100] Weibel ER (2017). Lung morphometry: the link between structure and function. Cell Tissue Res.

[CR101] Weibel ER, Taylor CR, Hoppeler H (1992). Variations in function and design: testing symmorphosis in the respiratory system. Respir Physiol.

[CR102] West MJ, Slomianka L, Gundersen HJ (1991). Unbiased stereological estimation of the total number of neurons in thesubdivisions of the rat hippocampus using the optical fractionator. Anat Rec.

[CR103] Willführ A, Brandenberger C, Piatkowski T, Grothausmann R, Nyengaard JR, Ochs M, Mühlfeld C (2015). Estimation of the number of alveolar capillaries by the Euler number (Euler-Poincaré characteristic). Am J Physiol Lung Cell Mol Physiol.

[CR104] Wulfsohn D, Knust J, Ochs M, Nyengaard JR, Gundersen HJ (2010). Stereological estimation of the total number of ventilatory units in mice lungs. J Microsc.

